# Third-Trimester Glucose Homeostasis in Healthy Women Is Differentially Associated with Human Milk Oligosaccharide Composition at 2 Months Postpartum by Secretor Phenotype

**DOI:** 10.3390/nu12082209

**Published:** 2020-07-24

**Authors:** Jessica L. Saben, Ann Abraham, Lars Bode, Clark R. Sims, Aline Andres

**Affiliations:** 1 J.L.S. Scientific Consulting, L.L.C., Thornton, CO 80229, USA; jlsaben@gmail.com; 2 Arkansas Children’s Nutrition Center, Little Rock, AR 72202, USA; SimsCR@archildrens.org; 3 Department of Pediatrics and Larsson-Rosenquist Foundation Mother-Milk-Infant Center of Research Excellence (MOMI CORE), University of California San Diego, La Jolla, CA 92093, USA; annmary_101@yahoo.com (A.A.); lbode@health.ucsd.edu (L.B.); 4 Department of Pediatrics, University of Arkansas for Medical Sciences, Little Rock, AR 72205, USA

**Keywords:** insulin resistance, oligosaccharides, maternal obesity, human milk composition, sialylation, DFLac, 3′SL, DSLNT, LSTc, LSTb

## Abstract

Human milk oligosaccharides (HMOs) are bioactive molecules in human milk that play a critical role in infant health. Obesity and associated metabolic aberrations can negatively impact lactation and alter milk composition. Here, the relationship between maternal glucose homeostasis and HMO composition from 136 healthy women was examined. Maternal glucose homeostasis (fasting plasma glucose and insulin, homeostatic model assessment for insulin resistance, and insulin sensitivity index) was evaluated at 30 weeks of gestation in healthy women (body mass index = 18.5–35 kg/m^2^). Human milk samples were collected at two months postpartum. HMO concentrations were measured via high performance liquid chromatography. Women were categorized into “secretor” and “non-secretor” groups based on 2′-Fucosyllactose concentrations (<100 nmol/mL, non-secretor). Pearson’s correlation analysis and linear models were used to assess the relationships between maternal glucose homeostasis and HMO concentrations. In non-secretors, third trimester fasting plasma glucose and insulin were negatively associated with total HMO-bound sialic acid and concentrations of the sialylated HMOs 3′-sialyllactose and disialylacto-N-tetraose. In secretors, difucosyllactose and lacto-N-fucopentaose-II concentrations increased and sialyllacto-N-tetraose c and sialyllacto-N-tetraose b decreased as insulin sensitivity increased. This study is the first to demonstrate a relationship between obesity-associated maternal factors and HMO composition in both secretor and non-secretor populations.

## 1. Introduction

Human milk oligosaccharides (HMOs) are the third most abundant class of biomolecules in human milk after lactose and lipids, contributing to about 20% of total milk carbohydrates. Although most HMOs are not digested, HMOs play a critical role in infant health through prebiotic and immunomodulatory actions [1]. HMOs promote the colonization of beneficial microbes, such as beneficial *Bifidiobacterium* species in the infant gut, preventing the growth of other harmful bacteria [2]. HMOs also reduce the risk of infections, by acting as antimicrobials that can mimic structures of viral receptors, providing a competitive advantage in preventing viral attachment to intestinal cells [3]. Finally, HMOs have proven beneficial toward the protection from necrotizing enterocolitis [4] and can signal to intestinal epithelial cells influencing gene expression, gastrointestinal development, and dampening inflammation [5]. Environmental and/or maternal factors that influence biosynthesis and composition of HMOs could, in theory, have a profound impact on these beneficial aspects of HMOs for breastfed infants.

HMOs are produced in mammary epithelium during pregnancy [6] and continue to be produced through lactation via glycosyltransferases that add five key monosaccharides (glucose, galactose (Gal), N-acetylglucosamine (GlcNAc), fucose (Fuc), and N-acetylneuraminic acid (sialic acid (Sia)) to HMO core structures in a specific order [7]. HMOs are present in the maternal serum, with concentrations increasing as the pregnancy progresses [6]. During lactation, over 150 HMOs have been identified in human milk where the proportion of fucosylated, sialylated, and non-fucosylated neutral HMOs in term human milk was recently reported as 35–50%, 12–14%, and 42–55%, respectively [5]. The most influential maternal characteristic impacting HMO composition is the expression patterns of Secretor (Se) and Lewis (Le) gene alleles, which code for different fucosyltransferases. Fucosyltransferase 2 (FUT2) is the “secretor” enzyme that transfers fucose residues in α1,2 linkages and fucosyltransferase 3 (FUT3) is the “Lewis” enzyme that transfers fucose residues predominantly as α1,3/4 linkages, leading to different patterns of fucosylated HMOs based on gene expression [8]. Several studies have shown that various environmental factors (geographical location, season of delivery, and maternal diet) [9,10] and maternal characteristics (age, ethnicity, parity, and mode of delivery) [10,11], including pre-pregnancy body mass index (BMI), can also alter HMO composition. A low BMI has been shown to be negatively associated with HMO biosynthesis, where women with a BMI of 14–18 had significantly lower HMO concentrations than those with a BMI of 24–28 [1]. Other studies have shown small, but significant correlations of BMI or obesity and HMO concentrations [10,11], suggesting that more research is needed to fully understand the potential impact of maternal body composition and metabolic health status on HMO concentrations.

Pre-pregnancy BMI has been associated with changes in the macronutrient [12,13] and bioactive composition of human milk [12,14]. Obesity is often complicated by metabolic dysfunction, such as hyperglycemia, hyperinsulinemia, and insulin resistance. During pregnancy and lactation, these conditions are exacerbated as a normal adaptation to the metabolic demands of supporting offspring growth [15,16]. In a recent study, Jantscher-Krenn et al. reported an association between circulating HMO concentrations during pregnancy and maternal glucose metabolism in a cohort of overweight and obese women. Interestingly, they found that including concentrations of the HMO 3′-sialyllactose (3′SL) in a model that predicted the development of gestational diabetes mellitus significantly improved the area under the curve for the model, suggesting that HMO production during pregnancy may influence maternal glucose homeostasis [17]. Given the recent findings that HMO production begins as early as 10 weeks of pregnancy [6], it is plausible to hypothesize that maternal factors during pregnancy such as hyperglycemia, hyperinsulinemia, or insulin resistance may influence HMO synthesis all the way into lactation.

Human milk lactose is the main carbohydrate fuel source for breastfed infants, it regulates milk volume [18], and it is the major core structure required for HMO synthesis [19]. Mammary gland lactose production is dependent on glucose transported into mammary epithelial cells via the glucose transporter, GLUT1 [20], or glucose produced in mammary epithelial cells via hexoneogenesis [18]. Therefore, glucose homeostasis is critical to milk production. Although it was previously believed that insulin did not play a direct role in mammary gland function [21], more recent research has begun to demonstrate that the mammary gland is responsive to insulin signaling during pregnancy and lactation [22,23]. For example, several studies have shown that insulin is critical for milk protein synthesis [23,24]. Additionally, impaired insulin sensitivity has been hypothesized to play a significant role in obesity-induced delayed lactogenesis [24]. To date, the relationship between HMO composition in mature human milk and obesity-associated maternal factors such as hyperglycemia, hyperinsulinemia, and insulin resistance have not been explored.

The goal of this study was to examine the relationship between measures of maternal glucose homeostasis during pregnancy and HMO composition in mature human milk from women with non-secretor and secretor phenotypes. Herein, we describe the first report of associations between specific HMO compositions and maternal circulating glucose, insulin, and insulin sensitivity that differ depending on secretor status.

## 2. Materials and Methods

### 2.1. Participants and Study Design

**Figure 1:**
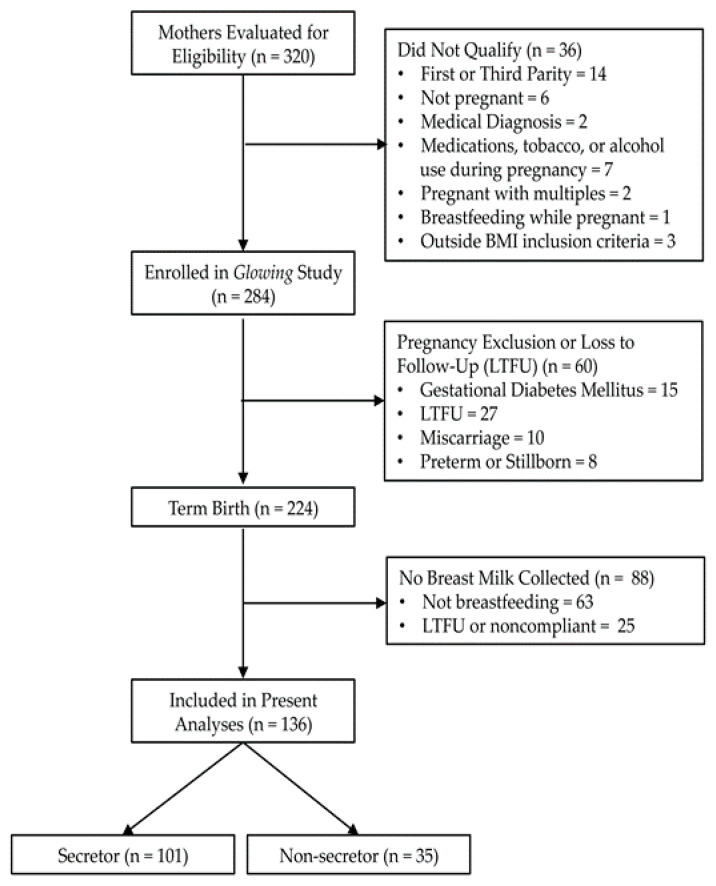
Study enrollment schematic. All breastfeeding participants enrolled in the *Glowing* study (www.clinicaltrials.gov, ID# NCT01131117) were eligible for analysis in the present study. Women were categorized into “secretor” and “non-secretor” groups based on the measured concentration of 2′FL (<100 nmol/mL, non-secretor).

A secondary analysis was performed on lactating participants (136 mother-child pairs) enrolled in the longitudinal *Glowing* study (www.clinicaltrials.gov, ID# NCT01131117) (Figure 1). Enrollment criteria have been previously described in detail elsewhere [25]. Briefly, women were eligible if they had a body mass index (kg/m^2^) between 18.5 and 35 kg/m^2^ (Figure 1). Women were excluded from the study if they had preexisting or ongoing medical conditions (e.g., diabetes mellitus, hypertension), chronic conditions requiring treatment, complications during pregnancy, a history of gestational diabetes mellitus, used medications during pregnancy that are known to influence fetal growth, smoke, or drank alcohol. Only second parity women who delivered healthy, full-term (≥37 weeks of gestation) children were included in the analyses. Women were categorized into “secretor” and “non-secretor” groups, based on the measured concentration of 2′-Fucosyllactose (2′FL) (<100 nmol/mL, non-secretor) [10]. The study procedures followed were in accordance with the ethical standards of the Institutional Review Board of the University of Arkansas for Medical Sciences.

### 2.2. Human Milk Collection and HMO Analysis

Participants were asked to collect a human milk sample at the second feeding of the day (or before 9 am) by fully expressing one breast at postnatal age 2 months (2M) to minimize diurnal as well as hind vs. fore milk variations. An aliquot (5–25 mL) was obtained after gently mixing the sample and frozen for future analyses. The samples were stored at −70 °C and shipped to the University of California (San Diego, CA, USA) (Dr. Lars Bode) for HMO analyses. Concentrations of HMOs were measured by high performance liquid chromatography on an amide −80 column (2 μm particle size, 2 mm ID, 15 cm length) with fluorescent detection as previously described [26]. The absolute quantification of the following 19 HMOs was determined using the non-HMO oligosaccharide raffinose as an internal standard added to all milk samples at the beginning of analysis: 2′FL, 3-fucosyllactose (3FL), (3′SL), 6′-sialyllactose (6′SL), difucosyllactose (DFLac), difucosyllacto-N-hexaose (DFLNH), difucosyllacto-N-tetrose (DFLNT), disialyllacto-N-hexaose (DSLNH), disialyllacto-N-tetraose (DSLNT), fucodisialyllacto-N-hexaose (FDSLNH), fucosyllacto-N-hexaose (FLNH), lacto-N-fucopentaose (LNFP) I, LNFP II, LNFP III, lacto-N-hexaose (LNH), lacto-N-neotetraose (LNnT), lacto-N-tetrose (LNT), sialyl-lacto-N-tetraose b (LSTb), and sialyl-lacto-N-tetraose c (LSTc). HMO Simpson′s diversity and evenness were calculated based on relative abundances of all annotated HMOs.

### 2.3. Maternal Body Composition and Gestational Weight Gain

Before or at 10 weeks of gestation, maternal weight and height were measured with a standing digital scale (Tanita Corporation, Japan) and a wall-mounted stadiometer (Perspective Enterprises, Portage, MI, USA), respectively. BMI was computed as kg/m^2^. Gestational weight gain was calculated based upon the weight gain between the participant′s first study visit and week 36 of gestation. The updated Institution of Medicine (IOM) guidelines for gestational weight gain based on BMI were used to evaluate the number of women categorized as having inadequate, appropriate, and excessive weight gain [27].

### 2.4. Metabolic Variables

A 100-g oral glucose tolerance test (OGTT) was performed after an overnight fast at gestational week 30, as previously described [28]. Blood was sampled from an antecubital vein (no anticoagulant) at baseline prior to glucose ingestion and at 30, 60, 90, and 120 min after glucose ingestion. Plasma glucose concentrations at the various time points were measured using an RX Daytona^®^ clinical analyzer (Randox Laboratories–US Limited, Kearneysville, WV, USA) and plasma insulin concentrations were measured using multispot assay kit (MesoScale Diagnostics, Rockville, MD, USA).

The updated homeostasis model assessment-2 calculator from the Oxford Centre for Diabetes, Endocrinology and Metabolism was used to estimate insulin resistance (HOMA-IR). OGTT-derived measurement of insulin sensitivity (estimated insulin sensitivity index (ISI, μmol*kg^−1^*min^−1^*pM^−1^)) was calculated using the Stumvoll et al. equation [28,29]. Fasting plasma insulin concentration, HOMA-IR, and ISI were used as indirect measurements of insulin sensitivity at 30 weeks of gestation.

### 2.5. Self-Reported Outcomes

At 2 weeks postpartum, mothers reported their delivery mode and infants′ sex. Gestational age was calculated using the mother′s last menstrual period and the child′s date of birth.

### 2.6. Statistical Analysis

Descriptive statistics (mean and standard deviation of the mean, median and Q1, Q3, and counts and percent) were calculated for demographic data, clinical characteristics, metabolic measures and HMO concentrations. Independent two-sample t-test and Pearson′s Chi-squared test were used to compare values between groups (non-secretor and secretor) for continuous and categorical data, respectively. Significance was set at *alpha* ≤ 0.05. Pearson′s correlations were used to determine the relationship between HMO concentrations and maternal fasting glucose or measures of insulin sensitivity (fasting insulin, HOMA-IR, and ISI). Linear models were constructed to determine maternal predictors of HMO concentrations. Independent models were constructed separately in secretor and non-secretor populations and included fasting glucose, fasting insulin, HOMA-IR and ISI as predictors of HMO concentrations. Other maternal characteristics including GWG, maternal age, and maternal race were included as covariates in each of these models.

## 3. Results

### 3.1. Participant Characteristics

Participant demographics and clinical characteristics are depicted in Table 1. About 74% of the total sample was phenotypically determined to be secretors, based on a human milk 2′FL concentration of > 100 nmol/mL at 2M postpartum. Overall, mothers were mostly Caucasian (87.5%) and around 30 years of age. The mean BMI was 25.483 kg/m^2^ and women gained on average ~12 kg over the course of pregnancy. The majority (42.5%) of women gained the appropriate amount of weight during pregnancy according to the IOM guidelines. Sixty-seven percent of women delivered via vaginal birth and the majority of infants were male, albeit only slightly the majority (56.6% vs. 43.4%, male vs. female, respectively). The non-secretor group averaged a significantly higher BMI (27.194 ± 4.048 kg/m^2^ vs. 24.890 ± 3.945 kg/m^2^, *p* = 0.004) and gave birth to a greater proportion of male infants (71.4% vs. 51.5%) compared to the secretors. All other demographic and clinical characteristics for the secretor and non-secretor groups did not differ between one another and were consistent with the whole population.

Fasting glucose and insulin levels and measures of insulin sensitivity (HOMA-IR and ISI) obtained at 30 weeks of pregnancy (Table 2) were all considered within normal range for pregnant women in their third trimester [15]. The average fasting insulin (62.500 ± 27.056 pmol/L vs. 42.719 ± 21.889 pmol/L, *p* < 0.001) and HOMA-IR values (1.179 ± 0.533 vs. 0.871 ± 0.446, *p* = 0.005) were significantly higher in non-secretors than in secretors (Table 2), whereas ISI values (0.049 μmol × kg^−1^ × min^−1^ × pM^−1^ ± 0.036 vs. 0.067 ± 0.035 μmol × kg^−1^ × min^−1^ × pM^−1^, *p* = 0.021) were lower, suggesting that the non-secretor population had decreased insulin sensitivity compared with the women who were secretors. This observations is likely a result of the greater proportion of overweight or obese women making up the non-secretor population (66.7%, Table 1) compared with those found in the secretor group (43.7%). Fasting glucose was similar between groups (*p* > 0.05).

### 3.2. HMO Composition at 2M Postpartum in Secretors and Non-Secretors

The HMO composition found in human milk collected at 2M postpartum averaged 16% sialyated, 73% fucosylated, and 13% neutral non-fucosylated HMOs based on the 19 HMOs (nmol/mL) measured in this study (Table 3). Secretors produced on average a 40% higher total HMO concentration compared to the non-secretors, which could be mostly attributed to the high production of 2′FL (median (Q1, Q3): 1580.40 (1143.25, 2156.02) nmol/mL vs. 6.40 (2.90, 11.50) nmol/mL, *p* < 0.001), LNFP1 (median (Q1, Q3): 1199.70 (746.20, 1854.05) nmol/mL vs. 142.80 (114.15, 167.10) nmol/mL, *p* < 0.001), and DFLNT (median (Q1, Q3): 1971.00 (1778.40, 2238.40) nmol/mL vs. 456.20 (290.60, 601.70) nmol/mL, *p* < 0.001) in the secretor group. Similarly, the median HMO diversity was ~40% lower in the non-secretor cohort than in the secretors and the total population. Non-secretor milk was characterized by significantly higher concentrations of 3FL (median (Q1, Q3): 4455.30 (3724.00, 5071.45) nmol/mL vs. 3320.10 (2459.20, 4384.90) nmol/mL, *p* < 0.001) and LNFPII (median (Q1, Q3): 2406.40 (2197.00, 2710.20) nmol/mL vs. 1679.40 (1341.45, 1986.12) nmol/mL, *p* < 0.001) compared to milk from the secretor group.

### 3.3. Measures of Glucose Homeostasis are Correlated with HMO Concentrations at 2M Postpartum

**Figure 2:**
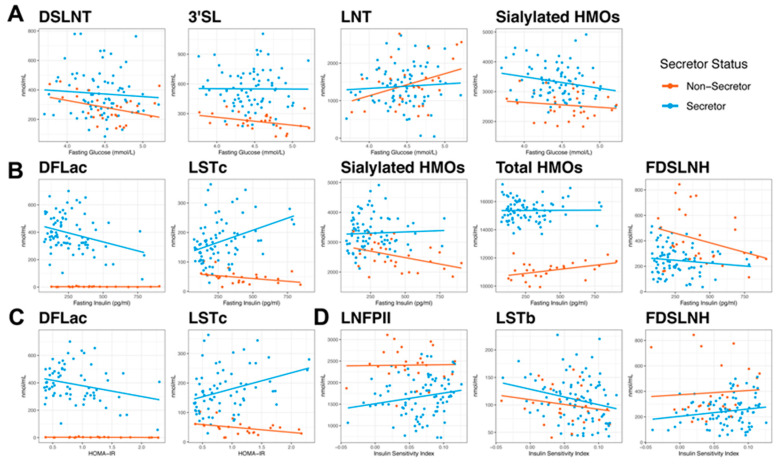
Correlations between HMO concentrations and measures of maternal glucose homeostasis. Maternal fasting glucose (**A**), fasting insulin (**B**), and insulin sensitivity measured via HOMA-IR (**C**) and insulin sensitivity index (**D**) were assessed at 30 weeks of pregnancy. Values were correlated with HMO concentrations measured in milk collected at 2 months postpartum from secretors (blue) and non-secretors (orange) to determine associations between maternal glucose homeostasis and HMO levels.

Significant correlations between fasting glucose at 30 weeks of pregnancy and HMO concentrations at 2M postpartum were only observed in the non-secretor group (Figure 2A and Table S1). Negative correlations between fasting glucose and human milk concentrations of DSLNT (R^2^ = 0.14, *p* = 0.048) and 3′SL (R^2^ = 0.15, *p* = 0.039) and a trending positive association with human milk concentrations of LNT (R^2^ = 0.12, *p* = 0.065) was observed in non-secretors. A trend toward a negative association between fasting glucose and total HMO-bound Sia (R^2^ = 0.045, *p* = 0.063) was observed in the secretor cohort. 

In human milk from non-secretor women, fasting insulin levels (Figure 2B and Table S2) were negatively associated with total HMO-bound Sia (R^2^ = 0.17, *p* = 0.027) but positively associated with total HMO concentrations (R^2^ = 0.18, *p* = 0.026) and there was a trend towards a negative association with FDSLNH concentrations (R^2^ = 0.1, *p* = 0.094). The FUT2-dependent HMO, DFLac, showed a negative association with fasting insulin (R^2^ = 0.098, *p* = 0.0043) and HOMA-IR (R^2^ = 0.072, *p* = 0.025) in the secretor group whereas the sialylated HMO, LSTc was positively correlated with fasting insulin (R^2^ = 0.13, *p* < 0.001) and HOMA-IR (R^2^ = 0.11, *p* = 0.006) levels in secretors (Figure 2B,C, respectively and Tables S2 and S3, respectively). Interestingly, non-secretors showed a negative correlation between HOMA-IR and LSTc concentrations (Figure 2C and Table S3, R^2^ = 0.16, *p* = 0.051), which was opposite from what was observed in the secretor population. Associations between HMOs and ISI (Figure 2D and Table S4) were only observed in the secretors, where concentrations of LNFP II were positively correlated and LSTb were negatively correlated with ISI (R^2^ = 0.058, *p* = 0.025 and R^2^ = 0.057, *p* = 0.024, respectively). There was also a trend towards a positive association between ISI and FDSLNH (R^2^ = 0.032, *p* = 0.091) in the secretor group (Figure 2D and Table S4).

### 3.4. Maternal Characteristics can Predict HMO Concentrations at 2M Postpartum

**Figure 3:**
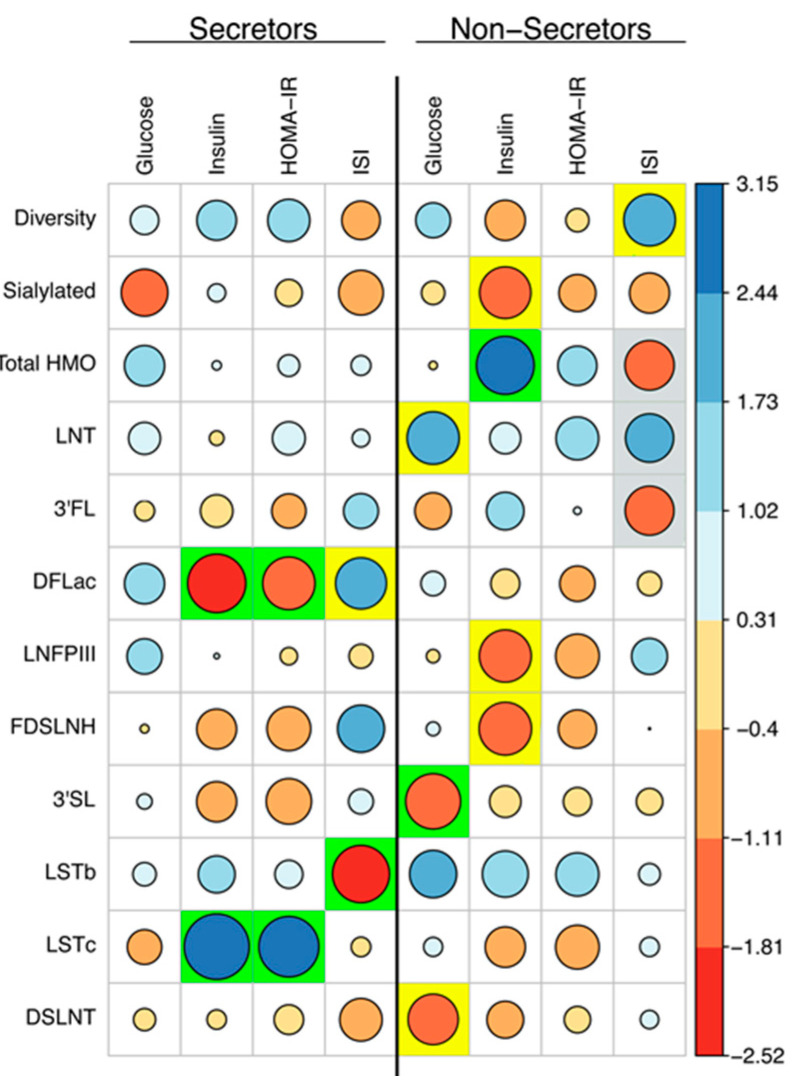
Linear models show significant relationships between maternal glucose homeostasis and HMO concentrations. Linear models were performed to determine the predictive association between fasting plasma glucose, fasting plasma insulin, HOMA-IR and ISI at 30 weeks of gestation and HMO concentrations in human milk at 2M postpartum after controlling for maternal gestational weight gain, maternal age, and maternal race. Circles represent the model test statistic where both size and color indicate the statistic value. Highlighted boxes indicated where the predictor was significant in the model. Green = *p* < 0.05, yellow = *p* < 0.075, and gray = *p* < 0.1.

Associations (test statistic for each linear model) between maternal characteristics and HMO concentrations are depicted in Figure 3. In secretors, maternal fasting glucose was not associated with HMO concentrations (Figure 3). Fasting insulin and HOMA-IR were negatively associated with DFLac concentrations (*β* = −0.230 nmol/mL, *p* = 0.014 and −71.902 nmol/mL, *p* = 0.046, respectively), whereas ISI tended to show a positive association with DFLac concentrations (*β* = 835.425 nmol/mL, *p* = 0.056) in secretors at 2M postpartum (Figure 3). Conversely, fasting insulin and HOMA-IR were positively associated with LSTc concentrations (*β* = 0.146 nmol/mL, *p* = 0.002 and 50.498 nmol/mL, *p* = 0.009, respectively) and ISI was negatively associated with LSTb concentrations (*β* = −313.351 nmol/mL, *p* = 0.017) in human milk at 2M postpartum (Figure 3). In the secretor group, maternal race had the greatest influence on individual HMO concentrations across all models compared to other maternal characteristics analyzed (Figure S1). Being non-Caucasian was associated with a number of sialylated HMO concentrations with higher 3′SL, 6′SL, LSTc, and LSTb (trend) and lower FDSLNH concentrations at 2M postpartum (Figure S1A). GWG also was associated with higher HMO-bound Sia, 3′SL, and DSLNH (trend) across most models (Figure S1C).

In non-secretors, maternal fasting glucose was negatively associated with human milk 3′SL concentrations (*β* = −87.735 nmol/mL, *p* = 0.034) and showed a tendency to predict lower DSLNT (*β* = −85.644 nmol/mL, *p* = 0.072) and higher LNT (*β* = 578.549 nmol/L, *p* = 0.054) concentrations at 2M postpartum (Figure 3). Fasting insulin levels at 30 weeks of gestation was associated with a 1.280 nmol/mL increase in total HMO levels for every 1 pg/mL increase of plasma insulin (*p* = 0.021) in non-secretors (Figure 3). Concentrations of LNFP III (*β* = −0.042 nmol/mL, *p* = 0.056), FDSLNH (*β* = −0.371 nmol/mL, *p* = 0.051), and total HMO-bound Sia (*β* = −0.807 nmol/mL, *p* = 0.062) tended to decrease with increasing levels of plasma insulin (Figure 3). HOMA-IR was not associated with HMO concentrations in non-secretors. ISI tended to be associated with increased diversity (*β* = 11.300, *p* = 0.060) and LNT (*β* = 6560.119 nmol/mL, *p* = 0.098) and decreased 3FL (*β* = −12,754.631, *p* = 0.090) and total HMO concentrations (*β* = −7770.195 nmol/mL, *p* = 0.084) in non-secretors (Figure 3). In the non-secretor group, maternal age and GWG had a greater influence on individual HMO concentrations than race. Across all models, maternal age was associated with decreased concentrations of DSLNH and 6′SL (Figure S1B), whereas GWG was significantly associated with increased concentration of 3FL and decreased levels of LNT, LFP I, and LFP III in most models (at least 3 out of 4) explored (Figure S1C). In secondary analyses, linear models were adjusted for maternal BMI, delivery mode or infant sex (Supplementary Table S1). Small changes were observed in the three models. While adjusting for maternal BMI (model B) lead to the highest number of changes in the results, caution should be taken when interpreting these results, as maternal BMI is highly collinear with the measures of maternal glucose metabolism.

## 4. Discussion

HMOs are major bioactive components of human milk and provide a number of advantages to infant health [1,30,31]. However, not all HMOs are created equal, when it comes to possessing beneficial and protective properties for the infant gut. Therefore, it is critical to elucidate factors that can influence HMO composition in order to find interventions that will help optimize early-life nutrition. Maternal genetics (FUT2 and FUT3 expression) have the greatest impact on HMO composition [32]. However to date, very few studies have reported on HMO concentrations in non-secretor populations most likely because non-secretors are the minority and many studies had small sample size to assess this group. In this study, several novel observations are presented that support our conclusion that maternal glucose homeostasis during pregnancy can significantly influence HMO composition in mature human milk. Notably, HMO composition was differentially associated with maternal characteristics depending on secretor status, highlighting the importance of discriminating effects based on secretor phenotype. Fasting plasma glucose levels were associated with overall concentrations of HMO-bound Sia and the concentration of 3′SL in non-secretors but no associations were observed between fasting glucose and HMO composition in the secretor population. For non-secretors, impaired insulin sensitivity was associated with an overall increase of HMOs concentrations whereas insulin sensitivity was associated with DFLac, LSTc, LSTb, and LNFP II concentrations in the secretor group. Together, these data are the first to show a relationship between obesity-associated maternal factors during pregnancy and HMO composition during lactation in both secretor and non-secretor populations.

The data presented in this study corroborate an association between maternal insulin sensitivity and the HMO composition of mature human milk. Insulin sensitivity is not only critical for glucose metabolism, but insulin resistance can increase various lipids [33] and alter amino acid metabolism [34] that may also impact HMO composition. Although a direct relationship between insulin sensitivity and HMO concentrations has not been demonstrated, it is reasonable to hypothesize that insulin signaling (either through direct effects on mammary epithelium or indirect effects on other organs) may contribute to mechanisms regulating HMO biosynthesis. To date, very little experimental data exists describing the biosynthesis of HMOs. However, because lactose is the most prevalent carbohydrate found in human milk and the reducing end of milk oligosaccharides contains lactose, it is assumed to be the initial substrate for HMO synthesis [19]. The most recent preclinical research on insulin signaling at the level of mammary epithelium has revealed that insulin is critical for stimulating gene expression that regulates mammary differentiation and milk lactose synthesis. Using mammary specific insulin receptor knockout mice, Neville et al. showed an insulin-dependent decrease in the expression of genes involved in milk lactose synthesis (among other processes) during pregnancy [22], confirming a direct role for insulin and the insulin receptor in secretory differentiation. Studies in mammary explants treated with or without insulin plus prolactin reported a 10-fold increase in alpha-lactalbumin and a three-fold increase in the beta-galactoside alpha-2, 6-sialytransferase gene expression when hormones were added [23]. Alpha-lactalbumin forms the regulatory subunit of lactose synthase and is critical for the production of lactose while beta-galactoside alpha-2, 6-sialytransferase encodes an enzyme that catalyzes the transfer of sialic acid from CMP-sialic acid to galactose-containing substrates. In the current study, total HMOs in non-secretors and a number of sialylated HMOs in secretors were associated with changes in maternal insulin levels and sensitivity. Insulin-dependent regulation of these genes and/or other enzymes critical for HMO biosynthesis may provide a potential link between maternal insulin signaling and HMO composition.

The association between insulin sensitivity during pregnancy and DFLac concentrations in mature milk in the secretor population was one of the strongest relationships observed in this study. In a recent explorative study, Larsson et al. observed a positive association between DFLac and infant weight velocity and DFLac and infant length at 5 months postpartum [35], suggesting a potential role for DFLac in promoting infant growth. They also found that concentrations of DFLac were significantly higher in human milk from mothers exclusively breastfeeding infants with excessive weight gain [35]. These findings are somewhat unexpected when considering the data presented herein which show maternal insulin resistance is associated with lower levels of DFLac in human milk at 2M postpartum, and the fact that maternal insulin resistance has historically been associated with an increased risk of obesity in offspring [16]. The differences in timing of these analyses could contribute to the observed discrepancy as HMO concentrations can change dramatically over the course of lactation [10,11]. More systematic studies that test the direct relationship between maternal insulin resistance, HMO concentrations, and infant growth are necessary to resolve this disagreement.

Sialylated HMOs have been linked to the protective effects of human milk against pathogenic infections and NEC. Recently, LSTc, LSTb, and DSLNT have been shown to express antimicrobial and antibiofilm activities against Group B *Streptococcus* [36]. In rodent models of NEC, the sialylated portion of DSLNT was the most critical for eliciting protective effects against death and NEC-induced pathologies [37], which was further corroborated in human cohort studies that showed that preterm infants who received milk with less DSLNT had a higher NEC risk [38]. Sialylated HMOs are also critical for infant growth [35,39,40]. In a recent study using two Malawian cohorts, Charbonneau et al. found lower sialylated HMO concentrations in human milk from mothers with growth-stunted infants [39]. Using preclinical models of undernutrition, they were able to establish a causal microbiota-dependent relationship between sialylated oligosaccharides and offspring growth via a greater ability to utilize nutrients for anabolism [39]. Furthermore, in a pilot study of exclusively breastfed infants, Larsson et al. demonstrated that concentrations of human milk 3′SL at 5 months postpartum were positively associated with infant length and concentrations of 6′SL were negatively associated with infant anthropometry (BMI-for-age z-score and fat mass index) in a secretor population [35]. Concentrations of DSLNT in human milk have also been shown to predict increases in infant fat mass [41]. A decrease in the overall sialylation of HMOs, as was observed in this study in non-secretors with increasing plasma glucose levels, may therefore result in decreased protection for the infant and potential adverse effects on infant growth. Conversely, women who were secretors and had decreased insulin sensitivity (defined by higher fasting plasma insulin, higher HOMA-IR, and lower ISI) were more likely to have greater amounts of LSTc and LSTb in their milk, demonstrating the need for mechanistic experimentation to elucidate the complexities of these relationships. However, with such compelling evidence to support a protective and growth-promoting role for sialylated HMOs, it seems imperative to identify the factors that influence the composition of sialylated HMOs in order to develop interventions that might enhance production. Lifestyle interventions that improve maternal glucose homeostasis may provide a realistic method for accomplishing this goal.

One of the most interesting secondary findings from this study was the stark difference in metabolic parameters (BMI and measures of insulin sensitivity) observed between non-secretor and secretor groups. Evidence suggests that the expression patterns of FUT2 (Secretor gene) and FUT3 (Lewis gene) can play a role in the predisposition to metabolic diseases [42]. Studies have suggested that a greater proportion of non-secretors exist within populations of patients with type-1-diabetes than within the general population [43,44], indicating a possible association between autoimmune disease and FUT2 expression. Women who were phenotypically characterized as non-secretors in the current study had significantly higher BMIs, fasting plasma insulin levels, and HOMA-IR scores with lower ISI values during pregnancy than did the secretors. These findings support the conclusion that FUT2 expression may be associated with the risk for metabolic syndrome in women, however additional studies designed to answer this specific question are necessary to further elucidate the relationship between FUT2 expression and predisposition to metabolic diseases.

Although this study has many strengths including: (1) the analysis of HMO concentrations in both secretor and non-secretor populations, (2) having a large sample size with well characterized participants, and (3) the use of multiple measures for glucose homeostasis to provide a comprehensive understanding of the relationship between HMO concentrations and glucose metabolism, it must also be interpreted within the context of its limitations. First, HMO concentrations change significantly over the course of lactation [10,11], and while this study examined a time point that represents mature human milk, it does not allow for the interpretation of HMO composition at other meaningful time points in lactation, such as in colostrum or when infants are transitioning to mixed feeding around six months postpartum. Secondly, linear models were deployed to explore the relationships between measures of maternal glucose homeostasis and HMO concentrations, and while these models controlled for a number of maternal characteristics strengthening their validity, they do not provide information to support a direct effect of glucose or insulin on HMO biosynthesis or metabolism. Additional studies that mechanistically test this relationship are necessary to make these kinds of conclusions. Finally, although the data presented herein support the conclusion that altered glucose homeostasis is associated with changes in HMO concentrations, future studies are necessary to interpret how the observed changes in HMO composition might impact infant health.

In conclusion, maternal obesity can impact both lactation and milk composition leading to greater risks in the postnatal period. However, obesity is complicated by a myriad of abnormal metabolic changes that may or may not play a role in how maternal obesity impacts human milk; making the path to finding interventions that could improve postnatal nutrition even more challenging. In this study, measures of maternal glucose homeostasis were differentially associated with HMO concentrations at 2M postpartum based on secretor phenotype, suggesting that hyperglycemia and insulin resistance that may contribute obesity-associated effects on human milk composition. HMOs provide advantages to breastfed and supplemented formula-fed infants through their protective and growth promoting properties. Therefore, HMOs make an ideal target for optimization in intervention studies.

## Figures and Tables

**Table 1:** Participant Demographics and Clinical Characteristics.

	Non-Secretor (*n* = 35)	Secretor (*n* = 101)	Total (*n* = 136)	*p*-Value
**Maternal Age (years)**				0.382 ^1^
Mean (SD)	30.182 (2.884)	30.743 (3.375)	30.599 (3.255)	
**Maternal Race (*n* (%))**				0.335 ^2^
Caucasian	29 (82.9%)	90 (89.1%)	119 (87.5%)	
Non-Caucasian	6 (17.1%)	11 (10.9%)	17 (12.5%)	
**Maternal BMI (kg/m^2^)**				0.004 ^1^
Mean (SD)	27.194 (4.048)	24.890 (3.945)	25.483 (4.084)	
**Maternal BMI ≥ 25.0 kg/m^2^**				0.011 ^2^
*n* (%)	24 (68.6%)	44 (43.6%)	68 (50.0%)	
**Gestational Weight Gain (kg)**				0.114 ^1^
Mean (SD)	11.050 (4.127)	12.248 (3.721)	11.955 (3.852)	
**IOM GWG Recommendation (*n* (%))**				0.819 ^2^
Inadequate	8 (22.9%)	25 (25.3%)	33 (24.6%)	
Appropriate	14 (40.0%)	43 (43.4%)	57 (42.5%)	
Excessive	13 (37.1%)	31 (31.3%)	44 (32.8%)	
**Gestational Age (weeks)**				0.996 ^1^
Mean (SD)	39.298 (0.813)	39.297 (0.904)	39.297 (0.879)	
**Delivery Mode**				0.864 ^2^
C-Section	11 (31.4%)	33 (33.0%)	44 (32.6%)	
Vaginal	24 (68.6%)	67 (67.0%)	91 (67.4%)	
**Infant Sex**				0.040 ^2^
Female	10 (30.6%)	49 (48.5%)	60 (43.4%)	
Male	25 (71.4%)	52 (51.5%)	79 (56.6%)	

^1^ T-test, ^2^ Pearson′s Chi-squared test.

**Table 2:** Maternal Glucose Metabolism at 30 Weeks of Pregnancy.

	Non-Secretor (*n* = 35)	Secretor (*n* = 101)	Total (*n* = 136)	*p*-Value
**Fasting Glucose (mmol/L)**				0.096 ^1^
Mean (SD)	4.553 (0.366)	4.435 (0.307)	4.466 (0.326)	
**Fasting Insulin (pmol/L)**				<0.001 ^1^
Mean (SD)	62.500 (27.056)	42.719 (21.889)	47.521 (24.628)	
**HOMA-IR**				0.005 ^1^
Mean (SD)	1.179 (0.533)	0.871 (0.446)	0.953 (0.488)	
**Insulin Sensitivity Index (μmol × kg^−1^ × min^−1^ × pM^−1^)**				0.021 ^1^
Mean (SD)	0.049 (0.036)	0.067 (0.035)	0.063 (0.036)	

^1^ T-test.

**Table 3:** Human milk oligosaccharide (HMO) Concentrations (nmol/mL) at 2 Months Postpartum.

	Non-Secretor (*n* = 35)	Secretor (*n* = 101)	Total (*n* = 136)	*p*-Value ^1^
**Fucosylated,** Median (Q1, Q3)				
**2′FL**	6.40 (2.90, 11.50)	1580.40 (1143.25, 2156.02)	1264.10 (19.95, 1914.35)	<0.001
**DFLac**	1.90 (0.85, 3.05)	358.50 (288.85, 469.75)	320.90 (147.40, 431.45)	<0.001
**LNFP I**	142.80 (114.15, 167.10)	1199.70 (746.20, 1854.05)	859.70 (197.20, 1513.12)	<0.001
**3FL**	4455.30 (3724.00, 5071.45)	3320.10 (2459.20, 4384.90)	3689.15 (2631.80, 4639.85)	<0.001
**LNFP II**	2406.40 (2197.00, 2710.20)	1679.40 (1341.45, 1986.12)	1814.00 (1458.40, 2249.90)	<0.001
**LNFP III**	28.30 (17.95, 44.15)	49.10 (36.40, 68.85)	42.90 (30.60, 65.20)	<0.001
**DFLNT**	456.20 (290.60, 601.70)	1971.00 (1778.40, 2238.40)	1842.80 (816.93, 2119.85)	<0.001
**FLNH**	135.55 (86.75, 189.23)	171.20 (124.60, 248.53)	167.55 (115.45, 235.67)	0.0501
**DFLNH**	25.25 (19.80, 37.58)	79.00 (50.55, 125.80)	56.80 (31.90, 99.40)	<0.001
**Sialylated,** Median (Q1, Q3)				
**3′SL**	223.90 (176.55, 275.95)	531.60 (408.00, 678.90)	428.35 (257.40, 608.32)	<0.001
**6′SL**	583.65 (436.90, 706.85)	770.90 (604.70, 997.42)	712.85 (574.25, 926.15)	<0.001
**LSTb**	95.45 (75.72, 127.78)	109.50 (80.95, 136.35)	107.50 (80.00, 136.10)	0.2361
**LSTc**	47.60 (31.50, 60.30)	166.75 (126.03, 215.65)	131.50 (73.95, 195.90)	<0.001
**DSLNT**	245.50 (214.60, 324.15)	353.70 (273.85, 476.07)	323.50 (245.50, 431.80)	<0.001
**DSLNH**	79.40 (57.65, 108.30)	136.40 (88.90, 182.30)	120.85 (78.72, 168.97)	<0.001
**Sialylated & Fucosylated,** Median (Q1, Q3)				
**FDSLNH**	304.50 (260.25, 558.40)	232.60 (152.70, 303.10)	256.75 (172.92, 346.80)	<0.001
**Non-fucosylated, neutral,** Median (Q1, Q3)				
**LNT**	1389.80 (876.35, 1647.30)	1396.70 (1061.00, 1776.18)	1396.30 (1016.25, 1738.75)	0.8131
**LNnT**	92.80 (61.65, 157.10)	101.90 (73.95, 145.40)	101.10 (71.55, 146.12)	0.5361
**LNH**	127.05 (75.15, 208.08)	173.65 (120.05, 258.90)	169.20 (104.90, 250.60)	0.0521
**Diversity and Evenness ^2^,** Median (Q1, Q3)				
**Diversity (log)**	1.47 (1.33, 1.62)	2.04 (1.89, 2.15)	1.97 (1.65, 2.13)	<0.001
**Evenness (log)**	−1.48 (−1.61, −1.32)	−0.91 (−1.05, −0.79)	−0.98 (−1.30, −0.81)	<0.001
**Total Concentrations** Median (Q1, Q3)				
**HMO-bound Sia**	2483.40 (2195.40, 2790.30)	3201.60 (2908.05, 3730.15)	3036.60 (2658.15, 3458.70)	<0.001
**HMO-bound Fuc**	8847.40 (8043.10, 9430.40)	13718.30 (12827.50, 14602.70)	13087.55 (10944.05, 14180.55)	<0.001
**Overall Total**	11180.15 (10737.27, 11533.83)	15407.05 (14860.42, 15806.28)	15008.35 (12668.05, 15657.68)	<0.001

^1^ Kruskal–Wallis rank sum test; ^2^ HMO Simpson′s diversity and evenness were calculated based on relative abundances of all annotated HMOs.

## Supplementary Materials

The following are available online at https://www.mdpi.com/2072-6643/12/8/2209/s1. Figure S1: The effects of maternal race, maternal age, and gestational weight gain on HMO concentrations, Table S1: Pearson′s Correlations Between Maternal Fasting Plasma Glucose (mmol/L) at 30 Weeks Gestation and HMO concentrations (nmol/mL) at 2M Postpartum, Table S2: Pearson’s Correlations Between Maternal Fasting Plasma Insulin (pg/mL) at 30 Weeks Gestation and HMO concentrations (nmol/mL) at 2M Postpartum, Table S3: Pearson′s Correlations Between Maternal HOMA-IR at 30 Weeks Gestation and HMO concentrations (nmol/mL) at 2M Postpartum, Table S4: Pearson′s Correlations Between ISI at 30 Weeks Gestation and HMO concentrations (nmol/mL) at 2M Postpartum.
