# Supplementary material for: Third-Trimester Glucose Homeostasis in Healthy Women Is Differentially Associated with Human Milk Oligosaccharide Composition at 2 Months Postpartum by Secretor Phenotype
Source: Nutrients. 2020 Jul 24;12(8):2209. doi: 10.3390/nu12082209 (PMC7468763; doi:10.3390/nu12082209)
Supplement: Supplementary file 1 [file nutrients-12-02209-s001.pdf]

Supplemental Table 1: Linear models were performed to determine the association between fasting plasma glucose, fasting plasma insulin, HOMA-IR and ISI at 30 weeks gestation and HMO concentrations in human milk at 2M postpartum after controlling for maternal gestational weight gain, maternal age, maternal race, maternal BMI, delivery mode and infant sex.

Maternal Glucose Metabolism	HMO	Secretor status	Model A[1]		Model B[2]		Model C[3]		Model D[4]	
Glucose	3'SL	NS	-87.735	0.034	**-79.262**	**0.075**	*NC*		*NC*	
Glucose	LNT	NS	578.540	0.054	**655.860**	**0.045**	*NC*		*NC*	
Glucose	DFLNT	NS	-152.992	0.235	*NC*		*NC*		**-210.67**	**0.093**
Glucose	LNH	NS	95.117	0.135	*NC*		*NC*		**121.689**	**0.049**
Glucose	Fucosylated	NS	-919.137	0.177	*NC*		*NC*		**-1,312.37**	**0.045**
Insulin	LNFPIII	NS	-0.042	0.056	**-0.038**	**0.103**	*NC*		*NC*	
Insulin	FDSLNH	NS	-0.371	0.051	*NC*		**-0.329**	**0.140**	*NC*	
Insulin	Sum	NS	1.280	0.021	*NC*		**1.031**	**0.106**	*NC*	
Insulin	Sialylated	NS	-0.807	0.062	*NC*		**-0.814**	**0.112**	*NC*	
ISI	Diversity	NS	11.300	0.060	**13.662**	**0.037**	*NC*		*NC*	
ISI	LNnT	NS	561.048	0.134	**720.213**	**0.072**	*NC*		*NC*	
ISI	Sum	NS	-7,770.20	0.084	*NC*		**-6,796.69**	**0.133**	*NC*	
ISI	LNT	NS	6,560.120	0.098	*NC*		**6,525.13**	**0.107**	*NC*	
ISI	DFLac	S	835.425	0.056	**589.976**	**0.247**	*NC*		*NC*	
ISI	LNFPII	S	2,734.199	0.064	**4,088.825**	**0.019**	*NC*		*NC*	
ISI	LSTb	S	-313.351	0.017	**-207.077**	**0.176**	*NC*		*NC*	
ISI	FDSLNH	S	575.621	0.106	**724.105**	**0.086**	**599.101**	**0.094**	*NC*	
HOMA-IR	DFLac	S	-71.902	0.046	**-61.270**	**0.101**	*NC*		**-70.480**	**0.052**

[1]Adjusting for maternal gestational weight gain, maternal age, and maternal race

[2]Adjusting for maternal gestational weight gain, maternal age, maternal race and maternal BMI

[3]Adjusting for maternal gestational weight gain, maternal age, maternal race and delivery mode

[4]Adjusting for maternal gestational weight gain, maternal age, maternal race and infant sex

HOMA-IR: Homeostasis Model Assessment of Insulin Resistance, ISI: Insulin Sensitivity Index, NC: No Change from Model 1, NS: Non-secretor, S: Secretor,
